# Counseling Patients on Preventing Prenatal Environmental Exposures - A Mixed-Methods Study of Obstetricians

**DOI:** 10.1371/journal.pone.0098771

**Published:** 2014-06-25

**Authors:** Naomi E. Stotland, Patrice Sutton, Jessica Trowbridge, Dylan S. Atchley, Jeanne Conry, Leonardo Trasande, Barbara Gerbert, Annemarie Charlesworth, Tracey J. Woodruff

**Affiliations:** 1 Department of Obstetrics, Gynecology, and Reproductive Sciences, University of California San Francisco, San Francisco, California, United States of America; 2 The American College/Congress of Obstetricians and Gynecologists, Washington, District of Columbia, United States of America; Roseville-Sacramento Kaiser Permanente, Sacramento, California, United States of America; 3 Pediatrics, Environmental Medicine, and Health Policy, New York University, New York, New York, United States of America; 4 Division of Behavioral Sciences, Professionalism, and Ethics, School of Dentistry, University of California San Francisco, San Francisco, California, United States of America; University of Cincinnati, United States of America

## Abstract

**Objective:**

Describe the attitudes, beliefs, and practices of U.S. obstetricians on the topic of prenatal environmental exposures.

**Study Design:**

A national online survey of American Congress of Obstetricians and Gynecologists (ACOG) fellows and 3 focus groups of obstetricians.

**Results:**

We received 2,514 eligible survey responses, for a response rate of 14%. The majority (78%) of obstetricians agreed that they can reduce patient exposures to environmental health hazards by counseling patients; but 50% reported that they rarely take an environmental health history; less than 20% reported routinely asking about environmental exposures commonly found in pregnant women in the U.S.; and only 1 in 15 reported any training on the topic. Barriers to counseling included: a lack of knowledge of and uncertainty about the evidence; concerns that patients lack the capacity to reduce harmful exposures; and fear of causing anxiety among patients.

**Conclusion:**

U.S. obstetricians in our study recognized the potential impact of the environment on reproductive health, and the role that physicians could play in prevention, but reported numerous barriers to counseling patients. Medical education and training, evidence-based guidelines, and tools for communicating risks to patients are needed to support the clinical role in preventing environmental exposures that threaten patient health.

## Introduction

Exposure to hazardous environmental chemicals, i.e., manufactured chemicals and metals, is linked to adverse health outcomes across all stages of the human life cycle including fertility, conception, pregnancy, child and adolescent development, and adult health [1]–[5]. Human exposure to environmental chemicals is ubiquitous. A population-based study found that virtually all pregnant women in the U.S. had measureable levels of at least 43 different environmental chemicals in their bodies, including chemicals that were measured at levels similar to those associated with adverse developmental and reproductive health outcomes in epidemiologic studies [6]. There are currently over 80,000 chemicals in commerce [7], [8], and exposure occurs through air, water, food and consumer products in the home and workplace. The majority of industrial chemicals have not been tested for potential reproductive/developmental harm [9].

Obstetricians are uniquely positioned to help prevent exposures to environmental chemicals with adverse developmental and reproductive health effects [2]. Pregnancy is a time when exposure to environmental contaminants can disrupt or interfere with the physiology of a cell, tissue, or organ [4], leading to permanent and lifelong adverse health outcomes that may be passed down to future generations [10]. Pregnancy is also an opportune time to prevent harmful exposures as it is a period when patient interest about health can be extremely high.

While obstetricians might play a role in preventing exposure to toxic chemicals among pregnant women, empirical evidence of whether or how obstetricians in the US are addressing the evidence that links environmental exposures to adverse reproductive health outcomes is lacking. Accordingly, we undertook the first study to examine attitudes, beliefs and practices among U.S. obstetricians about environmental exposures and their prenatal patients' health.

## Materials and Methods

The objective of this study was to assess the attitudes, beliefs and practices of obstetricians regarding prenatal environmental exposures through a mixed methods study including a national, quantitative survey of American Congress of Obstetricians and Gynecologists (ACOG) fellows and a qualitative focus group study of San Francisco Bay Area obstetricians. The protocol for this mixed methods study was approved by the University of California, San Francisco's Committee on Human Research.

### Quantitative Survey

We developed a 64-question survey to evaluate obstetricians' attitudes, beliefs, and practices related to environmental health which built upon on the survey of pediatricians developed and utilized by Trasande *et al* (2006) [11]. The adapted survey was pilot-tested and approved for use by ACOG.

ACOG is the largest U.S. organization of obstetricians with a membership exceeding 52,000, including over 28,000 “fellows.” ACOG fellows are physicians who are board certified in obstetrics and gynecology and are dues-paying members of the organization. All fellows were emailed the survey by ACOG in three “blasts” between September and November, 2011. The email message contained an invitation to participate in the study, a link to the online survey and an offer to enter into a raffle for an iPad as incentive for survey completion. Fellows were eligible to complete the survey if they were currently providing prenatal care.

Part 1 of the survey collected data on obstetricians' demographics and the characteristics of their patient populations. In part two of the survey, obstetricians rated statements about their attitudes, beliefs and practices around reproductive environmental health. We asked about the importance and impact of prenatal environmental exposures; the frequency with which they talked with their patients about environmental exposures; whether or not they counseled patients about 19 specific environmental exposures, their training in environmental health; and what sources of information they trusted about prenatal environmental exposures. We also asked about the choices they make in their own households regarding environmental exposures.

#### Statistical Analysis

Statistics were calculated using STATA 12 (StataCorp. 2011. *Stata Statistical Software: Release 12*. College Station, TX: StataCorp LP). The survey was conducted electronically, and data were directly imported and cleaned in STATA. In addition to descriptive statistics, we developed 3 scores—on the obstetricians' clinical practice, their beliefs, and their personal household practices—by averaging the results of the questions pertaining to each of the 3 categories. We compared scores among men and women and among age groups. We used a Wilcoxon rank-sum test to compare the obstetricians' confidence in taking a history on and discussing the impact of cigarette smoking with their patients compared to their confidence in discussing lead, pesticides, mercury and Bisphenol-A (BPA) exposures with their patients.

### Qualitative Focus Group Study

#### Study Participants

A convenience sample of obstetricians was drawn from a variety of settings in the San Francisco Bay Area, including private practice, academic health centers, county hospitals, and a large Health Maintenance Organization (HMO). We used publicly-available lists of local obstetricians as well as personal contacts, asking recipients to forward the message to colleagues. In collaboration with ACOG, we emailed invitations to all ACOG District IX (California) fellows.

Potential participants were screened for eligibility by telephone or email. Obstetricians were eligible if they currently saw pregnant patients in their practice, were ≥3 years post-residency training, and were either generalists or maternal-fetal-medicine specialists. Recruitment ceased when we signed up enough obstetricians for three focus groups with 6–10 participants per group.

#### Data Collection

The same person moderated every focus group. Sessions lasted approximately 90 minutes. Four investigators observed the focus groups and took field notes. Sessions were audiotaped and transcribed by a professional transcription service (PSF Transcription, San Francisco, CA). Transcripts were reviewed for accuracy by the same 4 investigators that observed the groups. Each participating obstetrician provided written informed consent and completed a written survey to collect demographic and medical practice information. Obstetricians were reimbursed $200 for participation in the focus group.

#### Data Analysis

Using principles of thematic analysis [12] and a subjective, interpretive “editing style” [13], we explored obstetricians' attitudes, beliefs, and practices regarding environmental exposures during pregnancy. Four investigators independently read transcripts, identifying prominent ideas and drafting preliminary coding categories. We then engaged in an inductive process of reading and manually coding transcripts, re-organizing coded data into themes. We reviewed results using memos to identify emerging themes and describe relationships among coding categories, as described in Miller and Crabtree (1999) [14]. The final coding scheme and analysis of the findings were reviewed and discussed until consensus was reached.

## Results

### Quantitative Survey

Of the 24,204 fellows that were on ACOG's email list and sent the email blasts, 2,624 responded and 110 were excluded because they were not currently seeing patients (n = 87) or they did not answer eligibility questions (n = 23). Table 1 presents demographic data on the 2,514 remaining obstetricians included in the analysis.

**Table 1:** Description of survey respondents (N = 2514).

Characteristic	No.	Percent
Age (mean ± SD)	50.6 (±9.0)	
Years in practice (mean ± SD)	18.8 (±8.9)	
Percent obstetrics (mean ± SD)	49.4 (±26.2)	
Sex		
Female	1,449	58.3
Male	1,037	41.7
Type of Practice		
Private not exclusively with HMO	1533	54.4
Public or community clinic	526	18.7
Teaching	385	13.7
Private exclusively with HMO	207	7.4
Other	111	3.9
Research	54	1.9
Patients on Medicaid		
0–25%	1,266	52.0
26–50%	665	27.3
51–75%	266	10.9
76–100%	236	9.7

Based on 2008 data from ACOG, approximately 75% of its fellows were practicing general obstetrician-gynecologists, obstetricians only, or maternal-fetal medicine specialists [15]. Therefore, we calculated that 18,153 ACOG fellows were eligible for the survey, giving us a response rate of 14%. Our findings represented practicing obstetricians from all 50 states and districts from across the country and the age and sex of our respondents corresponded with the statistics reported for ACOG at large in 2011 [16].

Comparing the difference of scores for clinical practice, beliefs, and personal practices among men and women we found that women had slightly higher scores than men (clinical practice p-value <0.0000; belief p-value = 0.001; personal practice p-value <0.0000); that is women tended to agree more with statements about environmental health and reported doing more to prevent exposures with their families. However we found no association with age and clinical practice, beliefs and personal practices.

Table 2 presents the survey results of obstetricians' self-reported attitudes, beliefs and confidence around environmental health. The majority agreed or strongly agreed that that conducting an environmental health history would help identify patient exposures (86%) and would help women prevent harmful exposures (80%). The majority (77%) of respondents also “strongly disagreed” or “disagreed” with statement that taking an environmental health history “would not be necessary”. Approximately half of the respondents “strongly disagreed” or “disagreed” that taking an environmental health history “would take too much time” (46%) and “would cause needless anxiety for patients” (45%).

**Table 2:** Obstetricians and gynecologists self-reported beliefs, attitudes and confidence around environmental. Health.

Attitude statements (n = 2505)	Percent	Mean ± SD
* Scale: strongly disagree (1); strongly agree (5)*		
Conducting and environmental health history would:		
Would identify exposures that my patients have been exposed to	86% agree or strongly agree	4.1±0.8
Would help women prevent exposures to environmental threats	81% agree or strongly agree	4.0±0.9
Take too much time	46% disagree or strongly disagree	2.8±1.2
Would cause needless anxiety for patients	45% disagree or strongly disagree	2.8±1.1
Would not be necessary	77% disagree or strongly disagree	1.9±0.9
**Belief statements (n = 2513)**	**Percent**	
* Scale: of little importance (1); of great importance (5)*		
The role of cigarette smoking during pregnancy	98% important or great importance	4.9±0.5
Assessing environmental exposures thought history taking	80% importan or great importance	4.2±1.1
The role of environmental exposures during pregnancy	71% important or great importance	4.1±1.2
**Confidence statements**		Mean ± SD
* Scale: strongly disagree (1); strongly agree (5)*		
Confidence in taking a history during prenatal care on: (n = 2506)		
Cigarette Smoking		4.9±0.4
Lead exposure		3.1±1.4*
Mercury exposure		3.1±1.4*
Pesticide exposure		2.9±1.4*
BPA exposure		2.1±1.3*
Confidence in discussing with prenatal patients the impact of: (n = 2507)		
Cigarette smoking on health		4.9±0.4
Lead exposure on health		3.4±1.4*
Mercury exposure on health		3.2±1.4*
Pesticide exposure on health		2.7±1.4*
BPA exposure on health		2.1±1.3*

*p<0.001 compared with lead with Wilcoxon Rank Sum test.

Seventy-eight percent of respondents reported they “strongly agreed” or “agreed” that they can reduce patient exposures to environmental health hazards by counseling patients and giving recommendations (mean ± SD = 2.1±0.9; 1 strongly agree – → 5 strongly disagree); yet half of the obstetricians surveyed (50%) reported they rarely (0–20% of the time) take an environmental health history (61% reported taking an environmental health history less than 40% of the time). Additionally, only 1 in 15 received training specific to taking an environmental health history.

**Figure 1:**
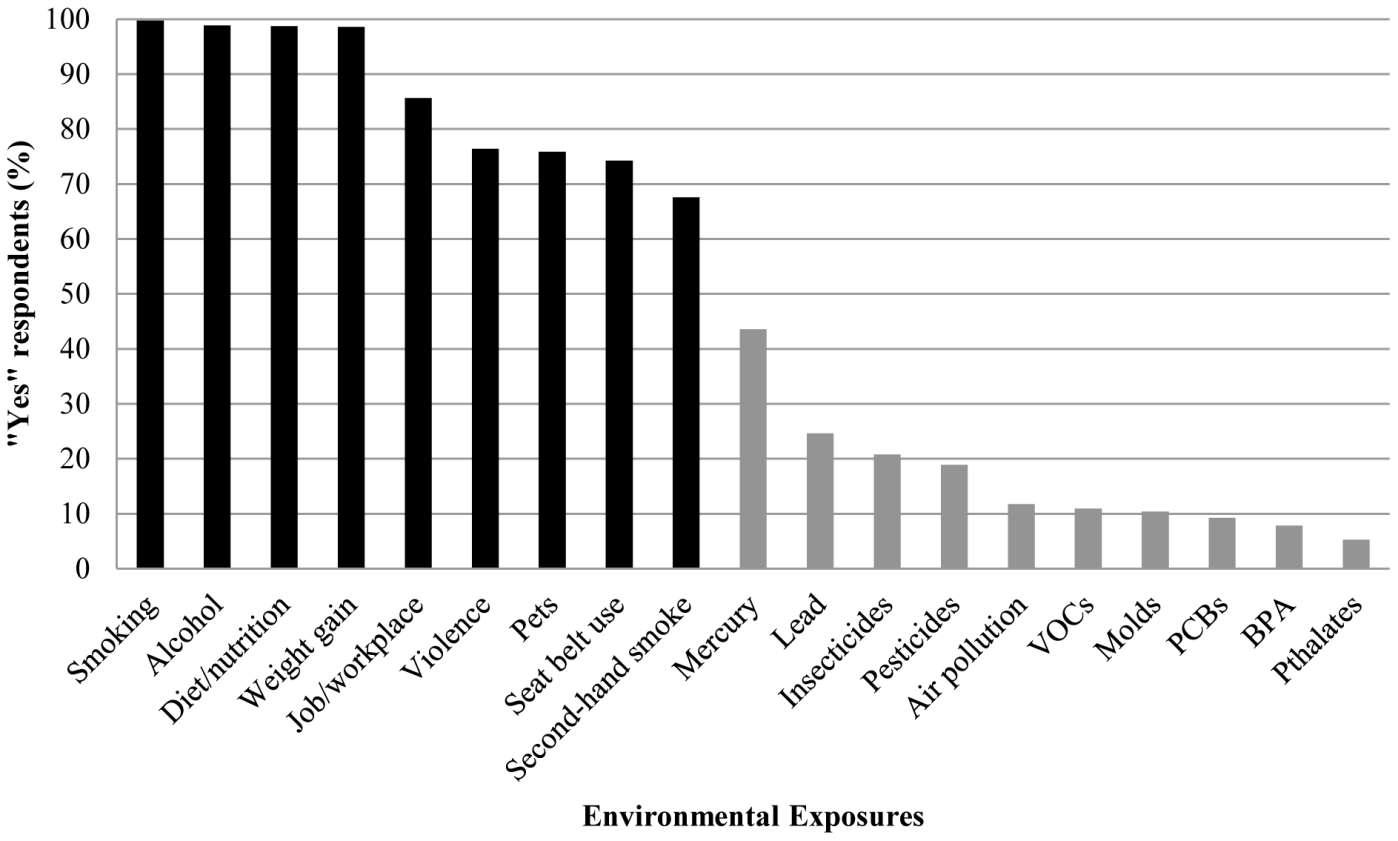
The percentage of “yes” respondents to the survey question, “Do you routinely discuss this issue as part of prenatal care?” Environmental exposures routinely discussed by less than 50% of survey respondents are shaded light grey. VOCs  =  volatile organic compounds; PCBs  =  polychlorinated biphenyls; BPA  =  bisphenol-A.

Virtually all respondents (99%) reported counseling prenatal patients about cigarette smoking, alcohol, weight gain, and diet/nutrition routinely; in contrast, less than 20% reported routinely counseling about environmental exposures commonly found in pregnant women in the U.S. such as phthalates, BPA, pesticides and PCBs (See Figure 1).

When asked to select resources they used for information concerning environmental exposures, 75% of obstetricians surveyed selected ACOG as a primary source of information and guidance, followed by government websites (58%) and the professional literature (57%). Likewise, 89% chose ACOG as the most helpful source for learning new information regarding prenatal health issues.

### Qualitative Study

We conducted three focus groups totaling 22 obstetricians between October of 2011 and January 2012. There were 20 female and 2 male obstetricians, and the mean age of participants for all three groups was 43 (range 32 to 63). Eight of the providers practiced in a public or community clinic, hospital or health center; four were private practice exclusively; six were private practice, but not exclusively HMO; one practiced in a teaching setting; and one practiced in a research setting. All participants were generalist OB/GYNs. The following key themes emerged in data analysis, and example quotes from the focus group transcripts are shown in Table 3.

**Table 3:** Focus Group Quotes.

**“Pandora's Box”**
“So it's a little bit complicated. It's a little scary barrel to open because I don't have an answer. I can say like, you know, “Yeah, I wouldn't want to work with that stuff all day either. But is it going to give your baby a problem, I don't know.””
“It throws you off track and I find with limited time it's better to stay on track and let them talk to the geneticist or the appropriate person. So I ask about exposure but not in such depth that my time with a patient is gone.”
“I think you always have to abstain from something that itself may produce a problem by causing anxiety and guilt potentially in the face of an adverse outcome. You have no idea what's really related to the exposure concern or not. And also, you have to weigh what is the alternative like switching a job, right? Or not using a certain thing and using another.”
**Uncertainty**
“I mean a lot of the time when you're talking to the person and they're sitting in the room and, you know, they eat at Popeye's four times a week, you have bigger fish to fry than some of this [environmental exposures], it seems, simply because we don't have enough information, you know? Because they'll have a BMI of 45 or something and you find out about their diet and the only time you have is if you spend it talking about [how] you really need to eat some other place and start exercising, you know?”
“There's sort of a million [exposures], if you think about all the potential exposures, but how many of them have really got a lot of proven concern that there's a big spike in problems because of anything, you know. So I'm trying to just limit the information, partly that I give to the patient, partly for myself.”
“I feel like there's a point in which we may make women just crazy with what they [have to avoid] without a lot of data.”
**Agency**
“It's a really complicated subject because I think there's a lot of class stuff in there. It's really hard to afford those choices. If I freak out like, ‘Whoa, look at that kid who's drinking Coke out of a BPA bottle’, it's like which one of those things should I deal with, right? Or do we just deal with, you know, wow, let's just talk about contraception or let's talk about your diabetes —prioritization is one of those really complicated things. And if I told my patients, ‘You know, you need to get rid of all of this stuff and you can't microwave this stuff,’ they might just look at me like I was a crazy person, you know?”
“I say [regarding] cosmetics, let them look up, do their own research on what they're asking to use. Or oftentimes I just tell them there's no data and that they have to make an educated decision and if they really can't stand their gray hair, they must dye their hair, then that's a decision they're choosing to do.”
“For patients who are very highly educated, I'd be able to say, “Okay, these are the things to think about, you know, watch your fish, watch your BPAs, obviously stay away from these things.” And she'd say, “Okay.” Because all of those words mean something to her, whereas if somebody with no medical literacy comes into the clinic, it's a whole different level of conversation. So I think any type of educational component has to be really geared to what a person's baseline—like you have to know how a baby is made to understand how chemicals may impact that.”
**Resources and Strategies**
“Well, obviously, something computer-based, you can push a button, you can find something really quickly and print something out for them. But the information has to come in two pieces, like what does it mean for you and what can you do about it if you want to avoid it.”
“I think if it comes out of an academic center, even if it's not evidence-based because the research hasn't been done, you could say, “Okay, so there's this substance and the concerns about it would be X, Y, or Z,” that's enough to pass on to a patient that might alter the patient's behavior towards, you know, exposure to that thing.”
“Yeah, my experience has been pretty reasonable to send them to genetic counselors, I mean, to some extent. I think a lot of it is the genetic counselors do have some data [about risks from exposures].”

#### “Pandora's Box”

Obstetricians feared broaching the topic of environmental health with patients, especially regarding chemicals other than lead and mercury. They felt they did not have adequate knowledge and understanding to answer patients' questions about exposures, and that this conversation would take time away from easier topics to address, like nutrition. The sheer number of chemicals in the environment was felt to be a barrier to addressing harmful exposures. Obstetricians were also concerned about harming patients by causing anxiety about exposures they could neither control nor understand; or making patients feel guilty if an adverse clinical outcome occurred.

#### Uncertainty

Participants expressed uncertainty regarding the degree of harm from environmental exposures because they do not feel confident in environmental health data in general, and/or because they believe that the evidence is inconclusive for particular exposures, and therefore not strong enough to merit action. With limited time for visits, participants prioritized obesity and diet counseling, which were felt to be more definitively linked to adverse health outcomes compared to environmental exposures.

#### Agency

This theme addressed “agency” i.e., the ability of patients to take action to reduce risk. This included concerns that patients, because of poverty, low literacy, and cultural factors, would be unable to take action to reduce harmful exposures. Participants struggled to help patients balance concerns about occupational risk with concerns about job loss and economic hardship. In contrast, providers with patients of higher socioeconomic status asked patients to do their *own* research and take ownership of their own choices.

#### Resources and Strategies

This theme considered the resources and strategies obstetricians used when counseling about prenatal environmental exposures. Participants preferred to refer to specialists including geneticists, or to an educational session in a class or group setting, rather than to counsel patients themselves. In each focus group, online resources were chosen as the most useful, but only if generated by a reliable authority such as ACOG or the Environmental Protection Agency (EPA). Concise printed materials with straightforward recommendations were also mentioned as a needed resource.

## Discussion

This is the first study to examine attitudes, beliefs and practices among U.S. obstetricians about prenatal environmental exposures. We found that obstetricians recognized the impact of the environment on reproductive health, but lacked training, time, and tools to take action to prevent harmful exposures. Survey participants believed that environmental exposures were important and that reproductive health professionals had a role in prevention. However, this concern did not translate into clinical practice. Few respondents reported routine counseling about exposure to environmental chemicals known to be harmful to reproductive health, and most felt ill-prepared to deal routinely with the issue.

Lack of certainty about the absolute and relative risk to patients from environmental exposures was a major factor in limiting obstetricians' ability to effectively counsel patients. Good clinical practice demands a level of scientific certainty and considers risks and benefits when advising patients about medical interventions. However, these hallmarks of clinical decision-making do not apply seamlessly to patients' environmental exposures. This is because, *in contrast to pharmaceuticals*, patient exposure to environmental chemicals generally occurs in the absence of evidence of safety and risk/benefit analyses [9], [17], [18]. Patient counseling for environmental exposures is comparable to a decision-logic for advising patients about taking a drug *prior* to its proven safety, where, in the absence of a compelling benefit, one would likely take a precautionary approach and recommend avoiding exposure.

In addition, there are inherent uncertainties in the evidence generated in environmental health science, which relies on toxicological non-human data and human observational studies. This type of scientific evidence is unfamiliar to, and/or perceived to be, “low quality” by physicians who consider randomized controlled trials (RCTs) as the “gold standard” evidence. However, ethics virtually precludes the use of RCTs in environmental health, and in vitro animal studies of reproductive and developmental toxicity serve as reliable predictors of human health effects [19]–[22], with studies showing that humans are at least as sensitive as the most sensitive animal species [21], [23].

Consistent with a “precautionary” approach to reducing risk in the absence of causal evidence, the Royal College of Obstetricians and Gynaecologists recently concluded: “Despite uncertainty surrounding the effects of common environmental chemicals, mothers should be made aware of the sources and routes of exposure, the potential risks to the fetus/baby and the important role that the mother can play in minimizing her baby's chemical exposure” [24]. They state that such information should be provided at obstetrical visits. Efforts are underway to create evidence-based guidelines by adopting the systematic and transparent methods empirically demonstrated as superior in the clinical sciences to the evidence that informs environmental health [25].

Another key finding is that over ½ of providers were concerned that raising the topic of environmental exposures to their pregnant patients would cause excessive anxiety and stress. This fear may be unwarranted, as biomonitoring studies have shown that women want to know and can react in a productive way to information about potentially harmful exposures [26]–[29]. However, these same studies also report that some women experience distress from the uncertainty of 1) the degree of harm caused by exposures and 2) a lack of information about how to reduce exposures. It thus seems logical that the focus in clinical counseling should be on the chemicals or substances that are most likely to cause harm *and* have practical strategies for reducing exposures on the individual level. More research studying pregnant women's responses to messages about environmental health would be helpful in designing counseling tools and interventions.

Our survey findings were limited by a low (14%) response rate and are not representative of all practicing U.S. obstetricians. Strengths of our survey findings include the fact that fellows from all U.S. states were represented in the sample, and our large sample size of over 2,000 respondents. Selection bias may have influenced the results if obstetricians interested in environmental health were more likely to participate. If so, our results would likely overestimate both the importance obstetricians place on environmental health and how often they counsel their patients about the topic.

The qualitative portion of our study was limited by small sample size and a single geographic region that may be more interested in environmental health than other locales. Accordingly, these findings are also not generalizable to all obstetricians across the U.S. However, the consistency between the responses of our Bay Area focus group participants and those of our national ACOG survey provided some validation of our qualitative results. Consistent with the goal of the survey, our findings do provide important insights into the attitudes, beliefs, and practices of U.S. obstetricians on the topic of prenatal environmental exposures that can be tested with more precision for training, regional, practice and other potential variation in future studies.

Our results were consistent with the fact that medical school and residency curricula do not typically include reproductive environmental health [2], [30]. Current efforts to include how environmental exposures impact patient health in curricula should be expanded, and CME should be provided to inform practicing obstetricians. Obstetricians in our study reported they needed evidence-based information to counsel patients, and that information vetted and supported by ACOG would be the most trusted. After our data were collected, ACOG released a Committee Opinion on “Exposure to Toxic Environmental Agents” that will be useful in guiding both policy and practice among obstetricians and other clinicians [31]. Many other professional societies and clinical organizations have recognized the impact that the environment can have on reproduction [32]. Our results underscore the role that health professional societies can play in operationalizing these statements, by providing trusted science-based guidelines for physicians and other reproductive health professionals.

Our focus group participants believed that their most vulnerable patients are less able to prevent exposures due to poverty, low literacy, and cultural factors. Women of lower socioeconomic status and women of color carry *greater* risk for environmental exposures [33]–[36], so it is concerning that the obstetricians in our study reported barriers to counseling those who are most vulnerable. Culturally and linguistically appropriate educational materials are needed to increase patient and community knowledge and awareness of the issue. Patient-centered brochures in English and Spanish that may help clinicians provide this important information to their patients can be downloaded at: http://prhe.ucsf.edu/prhe/toxicmatters.html.

Actions patients can take to reduce body burdens of some harmful substances include switching to organic food [37], [38], eating low-fat or non-fat dairy products and meat sparingly, avoiding fish high in mercury, and avoiding fast food and processed foods whenever possible [39], [40], Informed patients can also choose cheap, effective and less toxic alternatives for pest control and household cleansers. However, for certain routes of exposure, such as air and water pollution, individual action alone may be ineffective in preventing harmful exposures [41]–[43]. Additionally, women with occupational exposures have limited legal protections as regulations do not always reflect well-documented chronic health impacts. A 2007 study by the California Environmental Protection Agency found that 5 of 19 workplace chemicals known to cause reproductive or developmental harm lack a permissible exposure limit and 14 are not regulated as reproductive hazards [44]. Thus, physician and health professional societies can help reduce harmful exposures for pregnant women by lending their support for policy change.

In conclusion, U.S. obstetricians surveyed recognized some impact of environmental exposures on reproductive health, but lacked training, time, and tools to counsel patients. Incorporating ongoing scientific discovery into medical education and training, developing evidence-based recommendations for prevention, crafting effective and efficient tools for communicating uncertainty and risk to patients, and leveraging the voice of health professionals in policy arenas, are all essential strategies for reducing harmful environmental exposures and improving health outcomes.
